# Analysis of penicillin-binding proteins (PBPs) in carbapenem resistant *Acinetobacter baumannii*

**Published:** 2011-03

**Authors:** Jitendra Vashist, Vishvanath Tiwari, Rituparna Das, Arti Kapil, Moganty R. Rajeswari

**Affiliations:** *Department of Biochemistry, All India Institute of Medical Sciences, New Delhi, India*; * *Department of Microbiology, All India Institute of Medical Sciences, New Delhi, India*

**Keywords:** *Acinetobacter baumannii*, Bocillin FL, β-lactam resistance, penicillin-binding proteins

## Abstract

**Background & objectives:**

*Acinetobacter baumannii* is a Gram-negative, cocco-bacillus aerobic pathogen responsible for nosocomial infections in hospitals. In the recent past *A. baumannii* 0had developed resistance against β-lactams, even against carbapenems. Penicillin-binding proteins (PBPs) are crucial for the cell wall biosynthesis during cell proliferation and these are the target for β-lactams. Therefore, the present study was carried out to identify the PBPs in three (low, intermediate and high MICs) groups of carbapenem resistant isolates strains of *A. baumannii*.

**Methods:**

ATCC 19606 and 20 β-lactam resistant isolates of *A. baumannii* were obtained. Selective identification of the PBPs was done using Bocillin FL, a non-radioactive fluorescent derivative of penicillin.

**Results:**

The fluorescence emission from Bocillin-tag in SDS-PAGE gel of native strain identified eight PBPs, with apparent molecular weight of 94, 65, 49, 40, 30, 24, 22 and 17 kDa, however, these PBPs revealed alteration in carbapenem-resistant isolates.

**Interpretation & conclusions:**

A comparative analysis of PBPs in the resistant isolates with those of ATCC revealed a decreased expression of all PBPs except that of 65 and 17 kDa PBPs which were marginally downregulated, and simultaneous appearance of new 28 kDa PBP (in low and intermediate resistant isolates) and 36 kDa in high meropenem resistant group of *A. baumannii*. The present study indicated an association between alteration in PBPs and β-lactam resistance in *A. baumannii*.

*Acinetobacter baumannii* is an opportunistic, Gram-negative, cocco-bacillus which is known to cause nosocomial infections like septicaemia, bacteraemia, pneumonia, wound sepsis, endocarditis, meningitis, and urinary tract infections^1^–^3^. *A. baumannii* is resistant to desiccation and thus hard to eradicate once established in wards^4^,^5^. Carbapenem group, which includes imipenem and meropenem are last resort of β-lactams with highest efficacy and have broad spectrum against several Gram-negative bacteria including *A. baumannii*^6^.

Multidrug resistance in *A. baumannii* is a common clinical problem which further complicates the therapy^7^–^9^. The first known *A. baumannii* resistant to carbapenem was reported as early as in 1985 in Scotland^10^. The nosocomial outbreaks began in 1990s in all around the world and since then, incidence of carbapenem resistant isolates from hospitals worldwide has become a subject of concern^11^–^14^. In the past, multiple outbreaks of such infections caused by *A. baumannii* have been reported from different regions of India^7^,^15^,^16^.

Reports on resistance mechanisms in *A. baumannii* mainly focused on outer membrane impermeability^17^,^18^, production of beta lactamases^19^,^20^ and production of efflux pump^21^.However, equally important for bacterial survival are penicillin-binding proteins (PBPs) which play a crucial role in the synthesis of peptidoglycan, an essential component of the bacterial cell wall^22^. PBPs catalyze the final step of polymerization (transglycosylation) and cross-linking by transpeptidation of peptidoglycan. PBPs can be one of the most likely mechanisms for carbapenem resistance. Direct association of alteration in PBPs and their properties with β-lactam resistance was reported in various bacteria like *Escherichia coli* K-12^23^, *Pseudomonas aeruginosa*^24^ and *Streptococcus pneumoniae*^25^. It has been shown that modifications of PBPs play an important role in β-lactam resistance in Gram-negative bacteria^26^. Therefore, alterations in PBPs may play a role in drug resistance of *A. baumannii*. Although, the first carbapenem resistant strain of *A. baumannii* was reported more than 20 years back, till today there are just two reports, one from Spain^27^ and one from Germany^28^ which considered PBPs in resistance mechanism. Gehrlein *et al*^28^ identified seven PBPs in a imipenem sensitive clinical isolate (from Germany) and imipenem resistant (I^R^) clone of *A. baumannii* using radioactive ^14^C-penicillin. In the imipenem resistant (I^R^) clone of *A. baumannii*, they found a diminished expression of all the PBPs except 24 kDa PBP which is increased in its amount and showed low affinity for imipenem. Cuenca *et al*^27^ from University Hospital in Spain found large variations in the PBP patterns (analyzed by ^125^ I Ampicillin reagent) and found only 6 PBPs (93, 84, 73, 64, 49 and 38 kDa) with the decreased expression of 73 kDa PBP in *A. baumannii*. Both the reports utilized the radioactive material for identification of PBPs.

In the present study, an attempt was made to identify PBPs of *A. baumannii* in ATCC and resistant strains isolates obtained in a tertiary care centre in north India, associated with carbapenem resistance.

## Material & Methods

### 

#### Micro-organisms and growth conditions:

ATCC 19606 and 20 non repetitive β-lactam resistant isolates of *A. baumannii* were obtained from the Department of Microbiology, All India Institute of Medical Sciences (AIIMS), New Delhi, India during 2006 to 2008. Most of the clinical isolates were originated from ICUs of cardiology and neurology departments of AIIMS. All the clinical samples were inoculated on Mac Conkey agar at 37°C. Bacterial stocks were stored at -70°C in 60 per cent glycerol.

#### Materials:

Bocillin FL was purchased from Molecular Probes. Inc., USA, Luria-Bertani (L-B) broth and agar were from Hi-media, India, Tris-HCl, EDTA, SDS and protein molecular weight standards from Bio-Rad laboratories USA. N-lauroyl sarcosine and β-mercaptoethanol and α-cyano-4-hydrocinnamic acid matrix were from Sigma Chemical Co., USA. Mass spectrometry grade trypsin was from Promega, USA. All other chemicals obtained from Merck, India were of analytical grade. GeBA*flex*-tube kit used for protein elution was from Gene Bio-Application, Israel.

#### Antibiotic sensitivity tests:

Antibiotic susceptibility was evaluated via routine disc diffusion test, using discs impregnated with piperacillin (100 μg), ceftriaxone (30 μg), ceftazidime (30 μg), imipenem (10 μg), meropenem (10 μg), aztreonam (30 μg), amikacin (30 μg), netilmycin (30 μg), ciprofloxacin (5 μg), piperacillin-tazobactam (10 μg), cefaperazone-sulbactam (10 μg), and ticarcillin-clavulanic acid (10 μg). Minimum inhibitory concentrations (MIC) of all the 50 isolates for meropenem and penicillin were determined by agar dilution method^29^. The breakpoint concentration for the species was determined under the CLSI guidelines^30^. Twenty carbapenem-resistant isolates were used in the present study.

#### Identification of penicillin binding proteins using Bocillin FL:

Total membrane protein extraction from *A. baumannii* was done with a slight modification in previous method^17^,^18^. Bacteria were grown in 250 ml Luria-Bertani broth at 37°C for 19.4 h (overnight and constant time period) and harvested by centrifugation at 5000 g for 15 min. Pellet was resuspended in potassium phosphate buffer (20 mM, *p*H 7.5) and cells were disrupted by ultrasonication under cold conditions. The suspension containing the cell envelope was treated with DNase and subjected to ultracentrifugation for 30 min at 100000 X *g*. The pellet (total membrane fraction) obtained was washed and resuspended in 1 ml phosphate buffer (≈1 mg/ml). Approximately, 40 μg of the total membrane protein fraction was incubated with 5 μM Bocillin FL at 37°C for one hour. Later, a 2 per cent N-lauroyl sarcosine was added to this reaction mixture and incubated for one hour. Reaction mixture was subjected to ultracentrifugation (1, 00,000 X g) for 30 min and inner membrane fraction was separated as supernatant. Inner membrane protein fraction was then denatured with SDS-denaturing buffer at 100°C for 3-5 min; 20 μg of each reaction mixture was loaded on a 12 per cent SDS-PAGE. The protein gels were fixed using destaining solution immediately after electrophoresis and the protein bands were visualized under UV light (290 nm). The gels were photographed and the intensities were also measured using Bio-Rad gel Documentation. Isolation of proteins from each isolate was done three times and then the gel profiling was done for each extraction. Although it is well known that Bocillin binds selectively to PBPs only^31^,^32^, it was also checked whether Bocillin has any binding affinity with outer membrane proteins. To rule out this possibility, the total outer membrane extract was subjected with Bocillin and the resulting gel electrophoresis did not reveal any protein band confirming the fact that all those proteins detected by Bocillin in this study were only PBPs of inner membrane.

#### Protein elution:

Penicillin binding protein (40 kDa) of ATCC 19606 was extracted using gel-elution technique. A 40 KDa protein from 12 per cent SDS PAGE of ATCC membrane was manually cut. The gel slice containing the protein was transferred to GeBAflex-tubes in a horizontal electrophoresis tank filled with protein PAGE running buffer and electroelution was carried out at 80 volts for two hours. The electroeluted protein sample was lyophilized and further dissolved in 100 μl of distilled water. Homogeneity of the sample was checked once again on SDS PAGE.

#### MALDI-MS analysis of purified PBPs:

Purified protein of apparent molecular weight 40 kDa was excised from the gel followed by in-gel trypsin digestion. The sample preparation for mass spectrometry was done according to Wilm *et al*^33^. Matrix-assisted laser desorption ionization-time of flight (MALDI-TOF) mass spectrometry (MS) analysis was performed on a Bruker Daltonics GmbH spectrometer (Germany). Analyses were carried out using α-cyano-4-hydrocinnamic acid as a matrix. Monoisotopic peptide masses were assigned and database search was done using MASCOT v1.6b24 (Matrix Science, London, United Kingdom). The peptide mass tolerance was within 50 ppm.

## Results and Discussion

### 

#### Antimicrobial susceptibility and MIC:

The antibiotic susceptibility test data revealed that all the 20 isolates of *A. baumannii* obtained from samples from ICUs, were resistant to a large number of antibiotics which include more than 60 per cent of β-lactams and related compounds. Only combination drugs of β-lactams with β-lactamase inhibitors showed a slightly higher potency in antimicrobial activity, similar to that reported in *P. aeruginosa* and *A. baumannii*^34^. A slightly higher potency of the combination drug (β-lactam + lactamase inhibitor) suggested that some lactamases were also present in the resistant strain. The resistance against β-lactams is a multigenic factor and thus we approached the problem by studying PBPs for the account of resistance in the clinical isolates.

The MIC levels for meropenem in all the specimens of *A. baumannii* ranged from 4 to >64 μg/ml. The resistant isolates could be broadly classified under 3 categories on the basis of their MIC for meropenem (Table I). The less resistant group (G1) included six isolates that showed an MIC level less than 16 μg/ml. The intermediate resistant group (G2) included seven isolates with an MIC level ranging from 16 to 32 μg/ml and seven isolates of highly resistant group (G3) showed MIC levels >32 μg/ml. It is known that penicillin has very little effect on the newly emerged resistant strains of *A. baumannii*. MIC of penicillin was given along with MIC of meropenem for comparison purpose only.

**Table I:** MICs (μg/ml) of meropenem and penicillin for clinical strains of *A. baumannii*

Strain No. (source)	Grouping *	MIC for meropenem (μg/ml)	MIC for penicillin (μg/ml)

ATCC		< 1.0	1.0
119 (Blood)	G1	4	>512
96 (Blood)	G1	4	64
150 (Blood)	G1	4	>512
325 (Blood)	G1	8	>512
3/320(Respiratory tract)	G1	8	>512
355 (Blood)	G1	8	>512
170 (Blood)	G2	16	256
71 (Blood)	G2	32	>512
122 (Blood)	G2	32	>512
15/307 (Respiratory tract)	G2	16	256
25/350 (Respiratory tract)	G2	16	>512
15368 (Blood)	G2	32	64
15409 (Blood)	G2	32	64
168 (Blood)	G3	64	256
24/35 (Respiratory tract)	G3	64	128
721 (Blood)	G3	64	>512
688 (Blood)	G3	64	>512
398 (Blood)	G3	>64	>512
250 (Blood)	G3	>64	>512
17592 (Blood)	G3	64	>128

* MIC for meropenem: G1-≤. 8.0, G2- 16.0-32.0, G3- >32.0

#### Inner membrane proteins (IMPs) profiling:

**Fig. 1:**
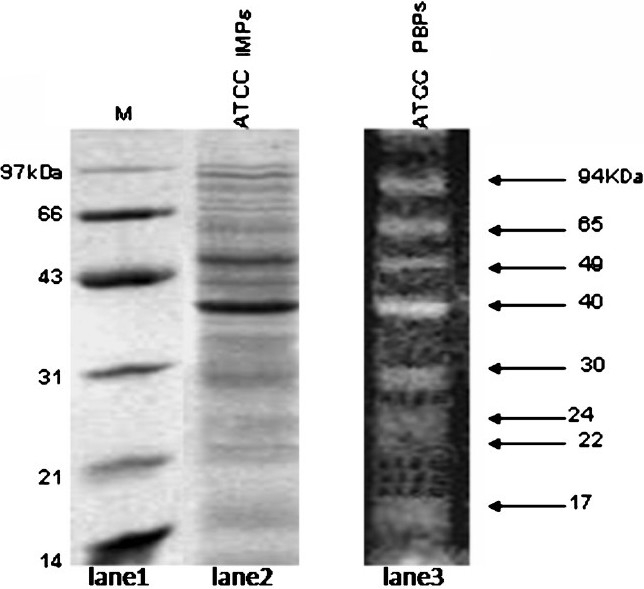
SDS-PAGE analysis of total inner membrane proteins of *A. baumannii* ATCC 19606. Lane 1, molecular weight marker; lane 2, total inner membrane proteins and lane 3, fluorescent image of Bocillin FL labeled-PBPs (from Fig. 4, lane 1).

Inner membrane consists of several proteins of signal transduction, electron transport system proteins, several transporters and PBPs, *etc*. PBPs are essentially located in the inner membrane; however, for detection of PBPs using Bocillin does not require inner membrane separation. To see how many of IMPs of *A. baumannii* are actually PBPs, the SDS PAGE profiles of IMPs were analysed (Fig. 1, lane 2). The total number of IMPs seen is quite large (>40 proteins).

#### Profiling of penicillin-binding proteins using Bocillin FL:

Protein profile of PBPs of wild type ATCC 19606 of *A. baumannii* and 20 resistant isolates were obtained by SDS-PAGE. The penicillin-binding proteins are conventionally detected on SDS-PAGE using radioactive Benzyl-C^14^ penicillin^23^. Detection methods using ^125^ I-labelled penicillin V and ^3^ H- or ^14^ C-labelled penicillin require days to week time^27^. The use of Bocillin FL offers several advantages over the radioisotope methods as it allows rapid detection up to 30 ng of a purified PBP under UV light and results can be obtained immediately after the SDS-PAGE. Detection of PBPs was done in a linear mode using 5 μM of Bocillin Fl which was much above the detectable concentration (0.8 μM) and much below the saturable concentration (25 μM) of Bocillin FL^31^,^32^.

PBPs in native strain:Before carrying out the PBP profiling on resistant isolates, the analysis on ATCC was done as a control. The analysis of the PBPs revealed eight bands in the wild type strain, which could be distinguished due to their well separated molecular weights (Fig. 1, lane 3).The eight PBPs had apparent molecular weights of 94 (PBP 1), 65 (PBP 2), 49 (PBP 3), 40(PBP 4), 30 (PBP 5), 24 (PBP 6), 22 (PBP 7), and 17 kDa (PBP 8). Gehrlein *et al*^28^ showed the presence of only seven PBPs in a laboratory-sensitive clinical isolate of *A. baumannii* using radioactive ^14^C-penicillin. In E. coli, a well documented Gram-negative bacteria; six PBPs in the range of 40-91 KDa were detected using Bocillin^31^.

Although one can see more than eight bands of PBPs, it is not possible to identify or resolve these bands separately. The relative expression levels of various PBPs in *A. baumannii* ATCC were quantitatively measured using densitometry. Of all eight PBPs, the PBP 4 (40 kDa) was highly expressed followed by the PBP 5 (30 kDa) and PBP 2 (65 kDa). The remaining PBPs were relatively less expressed. Although all the high molecular weight PBPs could be distinctly visualized under the UV light, PBP 6 and 7 with similar molecular weights (24 and 22 kDa, respectively) appeared as close bands and therefore were not clearly visualized on the gel.

**Fig. 2:**
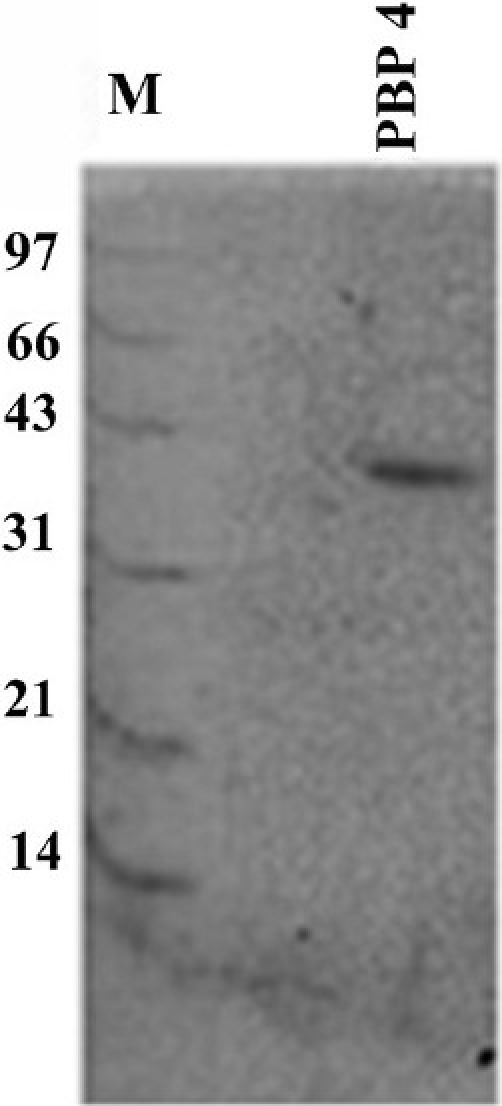
SDS-PAGE of purified 40 kDa protein (PBP4). Lane M is the molecular weight marker (in kDa). The purification was done by electro elution using GeBA*flex*-tube kit (Gene Bio-Application).

**Fig. 3:**
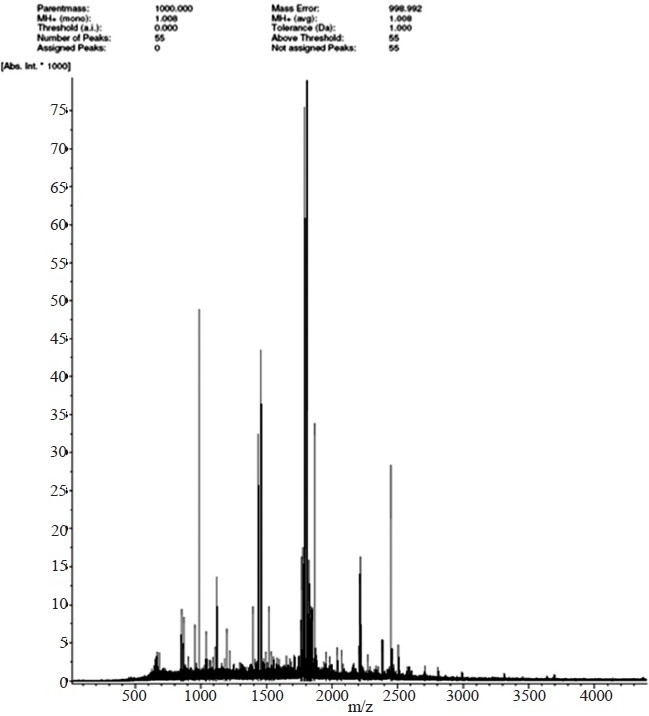
Peptide mass fingerprinting of 40 kDa protein of *A. baumannii* using Matrix-assisted laser desorption mass spectrometry (MALDI-MS). Peptide mass fingerprinting is done in positive mode (M+H^+^). X axis represents the molecular weight/charge of the marked peaks. Intensity (*y* axis) is in arbitrary units.

Purification and mass spectrometry analysis of 40 KDa PBP:The major protein, expressed in the native strain of *A. baumannii* with apparent molecular weight of 40 kDa was purified using the GeBA*flex*-tube kit. The purity of the protein was checked on 12 per cent SDS-PAGE which gave a prominent single band at 40 kDa (Fig 2). MALDI-TOF and peptide mass fingerprinting of purified protein is shown in Fig. 3. Of the 55 theoretical peptide masses obtained from mass spectrogram, 12 peptide masses on further analysis using MASCOT search showed identity with putative peptide sequences of PBP 2 (score 65%) of *Clorobium tepidium* (an anaerobic, green-sulphur bacterium) Table II. CLASTALW 2.0.12 analysis of PBP proteins of *A. baumannii* and *C. tepidium* showed high homology (Alignment score 32). Moreover, protein BLAST search of the three identified peptides (peptide masses 1393.674, 1789.845 and 1823.836) also showed the high homology with PBP2 protein (78, 88 and 71%) of *A. baumannii*. This further validated our result that the purified 40 kDa protein was a penicillin-binding protein.

#### Comparative analysis of PBPs in resistant isolates:

**Fig. 4:**
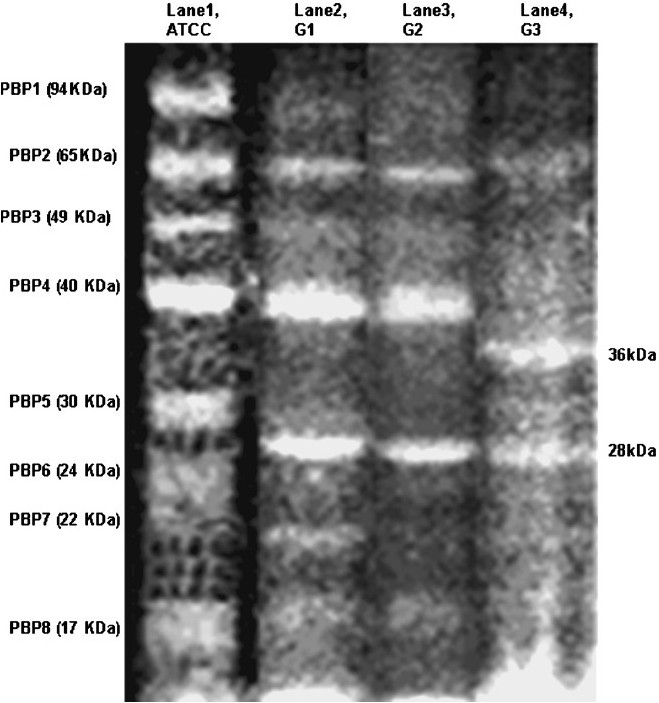
Comparative SDS-PAGE profile of penicillin binding proteins (PBPs) of ATCC and resistant isolates of *A. baumannii*using Bocillin FL. Lanes 1, 2, 3 and 4 represent PBPs of ATCC 19606, G1 (low resistant), G2 (intermediate resistant) and G3 (high resistant), respectively

Analysis of PBPs of resistant isolates was done according to their grouping based on their resistance towards meropenem. PBP profiling of all the 20 non repetitive isolates was done, and a representative profile of each group is presented in Fig. 4. t0 he PBP in resistant isolates were grossly similar with minor variations present in their relative expression as compared to those of ATCC. For example, in group G1 isolates, the percentage of densitometric values of PBPs ranged between 30-63 per cent (PBP1); 33-63 per cent (PBP2); 68-83 per cent (PBP3); 77-91 per cent (PBP4); 66-76 per cent (PBP5); 86-98 per cent (PBP6); 49-69 per cent (PBP7); 95-99 per cent (PBP8) with reference to the respective PBP expression in ATCC. These types of variations in PBP expression in clinical isolates have been also reported by other workers^27^.

**Table II:** Peptide sequences of PBP 40 kDa obtained from MALDIPMF using MASCOT showing similarity with PBP2 of *Clorobium tepidium*

Peptide molecular weight (>Da)	Sequence

950.482	IYEEELR
984.401	EGIDLPGER
1061.494	DLNEFVVAR
1393.674	AIQAVYPPGSTYK
1514.851	NKPKTQGADSTAVAK
1763.822	TAIDSTSTAPQSDLEGGD
1789.845	IFHDHGGRGHGIVNMK
1791.843	GVRYELVNPLGMLMGK
1807.837	MFGFGQREGIDLPGER
1809.842	IGFSGLERIYEEELR
1821.848	EQLDTLAEKGYTPDDK
1823.836	MFGFGQREGIDLPGER

PBPs were clearly visualized in all the 6 resistant isolates of Group 1 (MIC <16 μg/ml). It was interesting to note that all 8 PBPS were downregulated as compared to those of ATCC. The most characteristic feature of G1 group was the absence of PBP 5 (30 kDa) and simultaneous appearance of a new 28 kDa protein. Most of the expression of 94 kDa was diminished. This suggests that PBPs of 94 and 30 kDa are adversely affected in the initial stages of resistance.

The banding pattern in Group 2 was quite similar to that of Group 1, there was further decrease in the intensities of PBPs (Fig. 4, lane 3). The expression levels of 94 kDa PBP (~10%), 24 kDa PBP (~20%) and 22 kDa PBP (~30%) were very low as compared to the native strain.

The third group (G3) consisted of resistant isolates with very high MIC of meropenem (~64 μg/ml). The PBP profiles of this group showed a pattern different from that of ATCC profile. In general, Group 3 showed just three obvious bands with molecular weight 65, 36 and 28 kDa. Similarly large variations in PBP profiles was also reported earlier with downregulation of 73 kDa as seen in 65 kDa PBP of our hospital strains^27^. After high antibiotic load in *A. baumannii*, Gehrlein *et al* showed hyperproduction of 24 kDa, which was not observed in the present study^27^,^28^. Group 3 resistant isolates also displayed markedly diminished expression of the PBPs 94, 49, 24 and 22 kDa, making these practically absent (Fig. 4, lane 4). Most significant change noticed in Group 3 was manifestation of a 36 kDa PBP which might be a new PBP or altered product of a high molecular weight PBP. It is possible that conformation of the PBP with 36 kDa may not be conducive to accommodate carbapenem in resistant *A. baumannii*.

The bacteria can develop β-lactam resistance by altering their PBPs. To do this, the organisms can decrease their affinity to, β-lactams, overproduce a critical PBP, or produce a new or altered PBP. It is interesting to study the properties of 28 and 36 kDa proteins in order to understand structure-function relationship in β-lactam resistance of *A. baumannii*. Our study showed an association between deregulation of PBPs and the resistance in *A. baumannii*.
